# Comparison between Ultrasound Guided Transperineal and Transrectal Prostate Biopsy: A Prospective, Randomized, and Controlled Trial

**DOI:** 10.1038/srep16089

**Published:** 2015-11-03

**Authors:** Le-Hang Guo, Rong Wu, Hui-Xiong Xu, Jun-Mei Xu, Jian Wu, Shuai Wang, Xiao-Wan Bo, Bo-Ji Liu

**Affiliations:** 1 Department of Medical Ultrasound, Shanghai Tenth People’s Hospital, Tongji University School of Medicine, Shanghai, China; 2 Ultrasound Research and Education Institute, Tongji University School of Medicine, Shanghai, China; 3 State Key Laboratory of High Performance Ceramics and Superfine Microstructures, Shanghai Institute of Ceramics, Chinese Academy of Sciences, Shanghai, China

## Abstract

This prospective study of comparing transperineal prostate biopsy (TPBx) with transrectal prostate biopsy (TRBx) was aimed to provide evidence for clinicians to select the appropriate biopsy approach under different conditions. TPBx (n = 173) and TRBx (n = 166) were performed randomly for 339 patients who were suspicious of prostate cancer (PCa). The cancer detection rate (CDR), complication rate, visual analogue scale (VAS) score, most painful procedure, number of repeated biopsy and additional anesthesia, and operating time (starting from lying down on the operating table to getting up) were recorded. The results showed that TPBx and TRBx were equivalent in CDR (35.3% vs. 31.9%) and minor complication rate (44.9% vs. 41.0%) (both *P* > 0.05). The major complication rate was lower in TPBx than in TRBx (0.6% vs. 4.3%, *P* < 0.05). T*P*Bx was more time-consuming (17.51 ± 3.33 min vs. 14.73 ± 3.25 min) and painful (VAS score: 4.0 vs. 2.0); and it had higher rates of repeated biopsy (3.2% vs. 1.1%) and additional anesthesia (15.0% vs. 1.2%) (all *P* < 0.05). In summary, both TPBx and TRBx are effective to detect PCa. The major complication rate for TRBx is higher, whereas TPBx procedure is more complex and painful.

Prostate cancer (PCa) is the third leading cause of cancer-related death for men, accounting for 22.8% and 28% of all newly-diagnosed cancers in the male population in Europe and USA respectively^1^,^2^. Since Hodge *et al.*^3^ first introduced the systematic sextant biopsy protocol under transrectal ultrasound (TRUS) guidance, prostate biopsy has become a widely-accepted and routinely-performed technique to detect PCa^4^. The elevated serum prostate-specific antigen (PSA) level and abnormal digital rectal examination (DRE) finding are widely used to screen patients at a high risk of PCa^5^.

TRUS guided transperineal biopsy (TPBx) and TRUS guided transrectal biopsy (TRBx) are the two primary approaches to obtain prostate tissue for diagnosis of PCa. Their main differences lie in the puncture site, puncture route and the TRUS transducer. In TPBx, the needle punctures through skin at the perineum under the guidance of a bi-planar transducer; while in TRBx, the needle punctures through the anterior rectal wall under the guidance of an end-fire transducer.

Some studies have compared the two approaches from different perspectives. As to the cancer detection rate (CDR), TPBx is theoretically preferred for its better performance in sampling the peripheral and apical region where the PCa is supposed to be more common^6^. Early studies in the age of systematic sextant biopsy protocol suggested that TPBx seemed to be superior to TRBx^7^. However, with the increased number of biopsy cores, more and more studies argued that they were equivalent in CDR^8^,^9^. With respect to the associated complication, it seemed that no significant difference was present between them^10^. Nevertheless, it should be noted that infectious complications were more frequently observed for TRBx^11^.

Though the two approaches seem to have the same CDR and overall complication rate, it is interesting to note that TRBx is more popular globally^12^. Either American Urology Association or European Association of Urology recommends that TRBx is used as the most common method and TPBx is a useful alternative^13^. In a recent review, Chang *et al.* summarized some shortcomings of TPBx including consumption of longer time, training and financial constraints as well as the need for high-grade anesthesia^14^. Indeed, the above-mentioned factors might limit the use of TPBx; however, as far as we know, no prospective randomized controlled studies have been carried out to compare TPBx with TRBx on the above-mentioned factors directly. Therefore, with an aim to provide evidence for clinicians to select the appropriate biopsy approach under different conditions, this prospective randomized controlled study was performed.

## Methods

### End points

The primary end point of this prospective study was CDR. According to the World Health Organization Tumor Classification of Prostate, cores with acinar adenocarcinoma, neuroendocrine tumors, mesenchymal tumors or any other malignant findings are considered as positive results. On the other hand, pathological types including benign prostate hyperplasia (BPH), prostatitis, prostatic intraepithelial neoplasia (PIN), and atypical small acinar proliferation (ASAP) or any other non-malignant findings are considered as negative results. Furthermore, especially for acinar adenocarcinoma, the CDR was stratified by Gleason score for comparison according to the risk stratification schemes (i.e. Gleason score ≤ 6, =7 and ≥8) of European Association of Urology guidelines on prostate cancer^4^. In addition, the detection rate of very-low-risk (VLR) PCa (defined as Gleason score ≤ 6, ≤2 positive biopsies, PSA density < 0.15, ≤50% involvement on any core, and 12 or fewer cores sampled) that described by Epstein *et al.* was also investigated^15^.

The secondary end point was patients’ tolerance, with visual analogue scale (VAS; 0 = none to 10 = worst pain) used for pain level grading, and operating time (starting from lying down on the operating table to getting up) representing operating complexity. The investigation of complications was evaluated using Common Terminology Criteria for Adverse Events version 4.0 as a ref. 16. Overall, the mild complication was defined as “asymptomatic”, “self-limited” and “intervention not indicated”, while major complication was defined as “hospitalization”, “intervention indicated”, and even “life-threatening”.

### Patients

This comparative, prospective, parallel-group and randomized controlled trial was undertaken at a university-affiliated hospital in Shanghai, China. The Ethics Committee of the Tongji University monitored, reviewed and approved the study protocol. The study conformed to the standards of the Declaration of Helsinki. From June 2012 to Aug 2014, patients who were suspicious of PCa were included when meeting the following criteria: *(a)* PSA > 4.0 ng/ml for at least twice or *(b)* abnormal DRE findings. Among them, patients who met the following criteria were excluded: *(a)* > 80 years old, *(b)* unable to communicate effectively, *(c*) with a previous history of PCa or repeated biopsy, *(d)* with symptoms of urinary tract infection, acute urinary retention, and *(e)* coagulation disorders. All patients who agreed to be randomly assigned to the TPBx group or TRBx group gave written inform consent before enrollment. The protocol was registered at http://www.Clinicaltrials.gov, with the identifier of NCT01849835 on May 2013.

### Randomization and masking

The randomization procedure was carried out before biopsy using a computer-generated random-number sequence to assign patients to two groups. A fixed team made up of one urologist, one radiologist and one nurse performed all biopsy procedures. One pathologist with 20 years’ experience made all the pathological diagnoses. Besides, two independent investigators were in charge of the randomization procedure, data recording, and follow-up. All patients and investigators were aware of study group assignments except for the pathologist.

### Biopsy Procedure

The procedure of TRBx was in accordance with the “Evidence-based Guidelines for Best Practice in Health Care Transrectal Ultrasound Guided Biopsy of the Prostate (2011)”, and the procedure of TPBx was in accordance with routine practice reported in literatures^14^. Also, all biopsy procedures and relevant details were approved by the Clinical Research Steering Committee of the Tongji University.

Prior to biopsy, patients’ basic information and results of laboratory tests including blood routine examination, coagulation profile, urinalysis, PSA and fPSA level were collected. Patients who were treated with anticoagulation therapy were asked to stop relevant medications for 5 days before biopsy and for another two days after biopsy. All patients took ciprofloxacin (500 mg) orally twice a day for two days before biopsy. A cleaning enema was performed to each patient in the morning before biopsy. All biopsies were performed under real-time ultrasound guidance (EUB-6500; Hitachi Medical Systems, Japan), using a transrectal bi-planar transducer for TPBx and a transrectal end-fire transducer for TRBx respectively. The transrectal end-fire transducer was equipped with a special needle guide kit.

**Figure 1:**
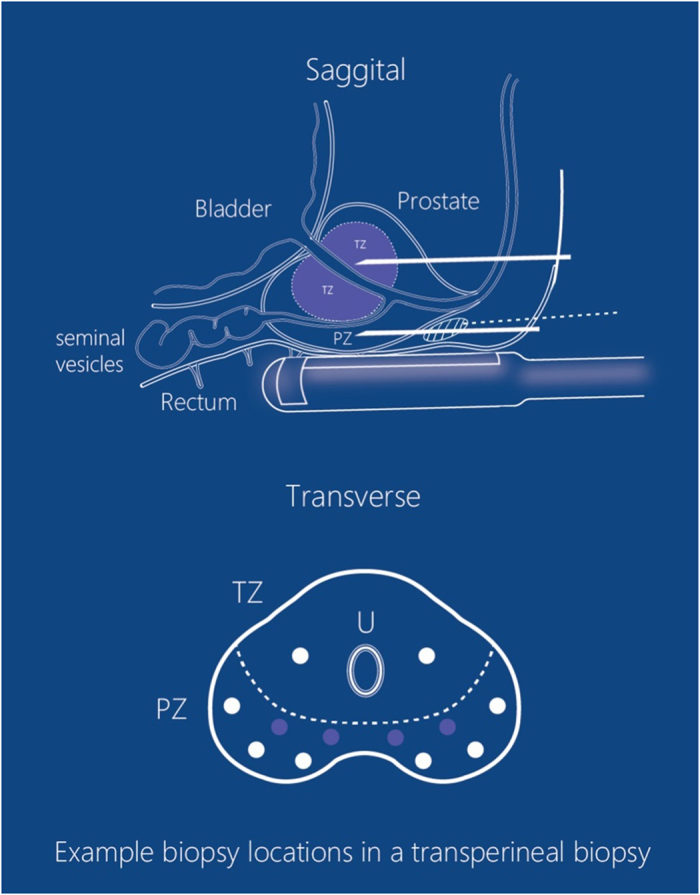
The protocol of TPBx. On sagittal section, both peripheral zone (PZ) and transition zone (TZ) biopsy are performed transperineally. The depots of PPNB are apical (in the white circle with diagonals), and the routes of administration are transperineal (white dotted lines). On transverse section, for 8-core TPBx, six cores are sampled from the PZ and the other two are from the TZ (marked as white circles). For 12-core TPBx, four more cores are sampled from the PZ (marked as violet circles).

**Figure 2:**
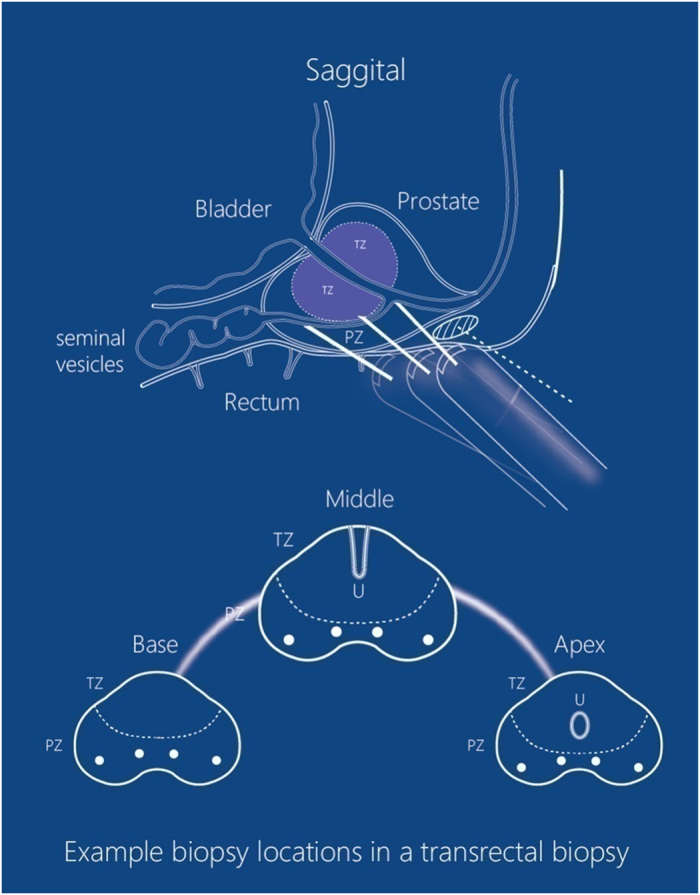
The protocol of TRBx. On saggital section, it shows that TRBx is performed in three planes of prostate (base, middle and apex plane). The depots of PPNB are apical (in the white circle with diagonals), and the routes of administration are transrectal (white dotted lines). On transverse section, it shows the protocol of biopsy in each plane (two parasagittal biopsies and two far-lateral biopsies). Four cores are sampled in base plane and four in apex planes respectively for 8-core TRBx, and another four cores are sampled in middle plane for 12-core TRBx.

TPBx procedures were performed with the patient in the lithotomy position, while TRBx procedures in the left lateral position. First, DRE was carried out to investigate the stiffness of prostate and the presence of prostate nodules. Subsequently, TRUS was performed to detect the PCa suspicious areas in the prostate and measure the prostate volume. The volume of the prostate was calculated using the formula: height × width × length × 0.52 (in ml). According to the recommendation of “Evidence-based Guidelines for Best Practice in Health Care Transrectal Ultrasound Guided Biopsy of the Prostate (2011)”, 12 cores were sampled from the prostate with volume > 50ml, whereas 8 cores from prostate with volume < 50 ml. Additional two cores were sampled from every suspicious area detected by TRUS or DRE. The protocols of the two procedures were shown in Figs 1 and 2. It should be emphasized that sampling of transitional zone (TZ) on initial biopsy did not conform to the guideline for TRBx procedure^17^. However, TZ biopsy was considered useful and performed in TPBx^18^. Therefore, we reserved TZ biopsy for TPBx in the present study.

Periprostatic nerve block (PPNB, 2% lidocaine, 10ml) was administered under ultrasound guidance (Figs 1 and 2). Additional PPNB (2% lidocaine, 2 ml) was administered at each puncture site where patient could not tolerate the pain. An 18-gauge automatic Magnum Biopsy gun (C R Bard, Inc., Covington, GA, USA) with a cutting length of 22 mm was used to obtain specimens. Each core was site-specifically labeled and was packed separately. According to the quality requirement^19^, if cores were fragmented, shorter than 10mm, or absent, another sampling would be performed at the same site. Failed cores would be recorded and counted into the total for analysis.

The biopsy procedure ended when patients got up from the operating table. Immediately, patients’ VAS score, number of additional anesthesia, causes of pain, and operating time were recorded. On the seventh day after biopsy, each patient was scheduled for a telephone review to assess possible complications. Long-term follow-up was performed on an “as-needed” basis. Any feedbacks that were confirmed relating with biopsy would be recorded. The procedure would be terminated if patients met any exclusion criteria or they requested to stop. The patient who did not complete biopsy procedure would not be withdrawn.

### Sample size estimation and Data analysis

Statistical analysis was performed using the SPSS 17.0 software (SPSS Inc. Chicago, IL, USA). The quantitative data were expressed as mean ± standard deviation when they were normally distributed, or expressed as median (inter-quartile range) when they were skewedly distributed. The proportions were given when appropriate. The CDR was used to estimate the sample size. At the beginning of this trial, a sample size of 100 (50 vs. 50 in each group) patients enabled a statistical power of 0.80 to detect a 4.1% difference of CDR (TPBx vs. TRBx = 31.7% vs. 27.6%). Moreover, findings in the previous literatures^14^ showed that the difference in CDR was not significant and was usually less than 10%. Thus, we assumed that a 10% difference between the two approaches was accepted as the margin in this non-inferiority test. It would require 264 patients for statistical power of 80% to detect this difference. Predicting a 10% exclusion rate, we planned to recruit at least 300 patients. Independent samples *t* test, Fisher’s exact test, and Wilcoxon Rank-Sum test were used to compare the two approaches when applicable. All statistical tests were two-sided, and a significant difference was considered when *P* < 0.05.

## Results

### Enrollment

**Figure 3:**
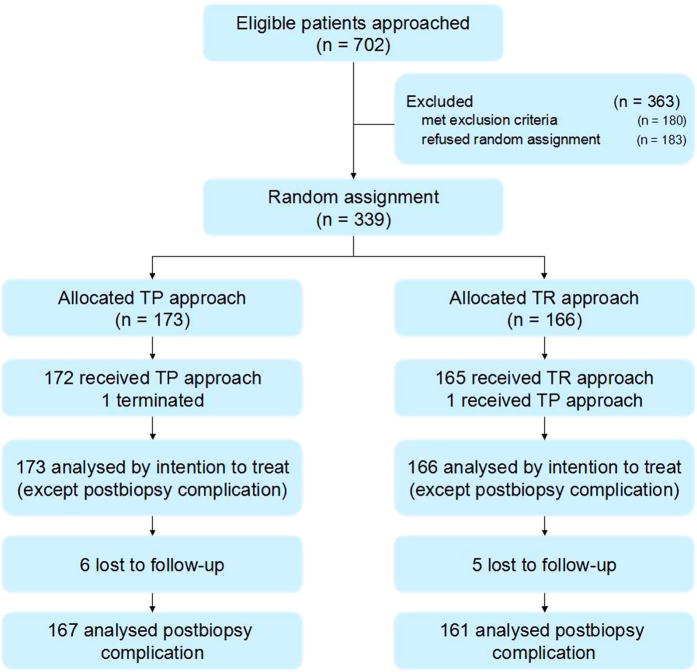
Trial profile.

702 patients were scheduled to undergo prostate biopsy (Fig. 3). Among them, 180 patients met the exclusion criteria and 183 refused to be assigned randomly. We anticipated trial end when 320 to 350 patients were recruited. Finally, a total of 339 patients agreed to participate in the trial. They were randomly assigned to the TPBx group (n = 173) and the TRBx group (n = 166).

### Basic characteristics

One patient from TPBx group terminated operation at the third puncture because of unbearable pain. This patient gave 10 points for the VAS grading, which was the highest among all patients. One patient from TRBx group was transferred to TPBx group because of continuous rectal bleeding, who were analyzed together in his originally assigned group using the intention-to-treat principle. The baseline characteristics and biopsy protocols of the patients from TPBx and TRBx groups were listed in Table 1. The above comparisons were generally comparable, except for the failed sampling cores. Among the failed sampling cores, a total of 62 cores (3.2%) were fragmented (n = 39), shorter than 10 mm (n = 5), or absent (n = 18) in the TPBx group; and a total of 20 cores (1.1%) were fragmented (n = 8), shorter than 10 mm (n = 5), or absent (n = 7) in the TRBx group. Further analysis revealed that the failed sampling cores were observed more frequently in the TPBx group (*P* < 0.001).

### CDR

A total of 114 patients (33.6%) were diagnosed with PCa. They were composed of 112 prostate adenocarcinomas (TPBx vs. TRBx = 61 vs. 51), one prostate rhabdomyosarcoma and one poorly differentiated carcinoma (both from the TRBx group) (Table 2).

Analysis of CDR showed an equivalent ability of the two approaches in detecting cancer. The positive rate was 35.3% vs. 31.9% (*P* = 0.566), and positive cores rate was 13.9% vs. 12.5% (*P* = 0.224) for the TPBx and TRBx group, respectively. Likewise, subgroup analysis showed that there was no significant difference in the proportion of each pathological pattern (all *P* > 0.05). Also, when CDR was stratified into three level by Gleason score, there was no significant difference in each one (all *P* > 0.05). There were six and five patients who met the criteria of VLR cancer being observed in the TPBx and TRBx group respectively. The proportion of VLR cancer was similar between the TPBx and TRBx groups (3.5% vs. 3.0%, *P* = 1.000).

### Complications

Six patients from the TPBx group and five from the TRBx group were lost to follow up respectively. Among the rest 328 patients (TPBx group = 167; TRBx group = 161), 45.5% and 45.3% of patients from TPBx and TRBx groups respectively claimed minor or major complications occurred (*P* = 0.912) (Table 3).

Overall, there was no difference between TPBx and TRBx groups in minor complication rate (44.9% vs. 41.0%, *P* = 0.504). In subgroup analysis, each patient with minor complications had one or more types of minor complications, including mild hematuria (19.8% in TPBx group vs. 23.0% in TRBx group), low fever (1.2% vs. 4.3%), mild rectal bleeding (0% vs. 8.7%), mild pain (35.3% vs. 13.0%), and others (6.6% vs. 7.5%). A significant higher rate of mild rectal bleeding in the TRBx group (*P* < 0.001) and a significant higher rate of mild pain in the TPBx group (*P* < 0.001) were observed. The incidence rates of other minor complications between the two groups were comparable.

Major complications occurred at a significantly lower rate of 0.6% in the TPBx group comparing to 4.3% in the TRBx group (*P* = 0.034), including high fever (0% in TPBx group vs. 1.2% in TRBx group), sepsis (0% vs. 0.6%), severe rectal bleeding (0% vs. 1.2%), and vasovagal event (0.6% vs. 1.2%).

### Operating time and VAS pain scores

The mean operating time of TPBx group was longer than that of TRBx group with statistically significant difference (17.51 ± 3.33 min vs. 14.73 ± 3.25 min, *P* < 0.001). The mean VAS pain score of TPBx group [4.0 (1.0–6.0)] was significantly higher than that of TRBx group [2.0 (0.0–4.0)] (*P* < 0.001) (Table 4).

Subgroup analysis showed that fewer patients did not feel pain during the whole procedure in the TPBx group (1.7%) compared with the TRBx group (22.3%). Fewer patients suffered from transducer insertion in the TPBx group (14.5%) compared with the TRBx group (42.2%). More patients in the TPBx group felt most painful during the procedure of anesthesia (63.6%) compared with the TRBx group (17.5%). Additionally, patients in the TPBx group (15.0%) required additional anesthesia more frequently than patients in the TRBx group (1.2%). All differences above were statistically significant (all *P* < 0.001). There were no significant differences in other procedures among the most painful procedures.

## Discussion

Systematic biopsy has been considered as the golden standard for PCa diagnosis and grading. In history, TPBx was performed in 1922, as the world’s first prostate biopsy^20^ and TRBx appeared 15 years later^21^. During those years, all prostate biopsies were performed without image guidance. It was not until 1989 that Hodge *et al.* started a new era of TRUS guided sextant prostate biopsy^3^. After further improvements of the end-fire transducer, TRBx became the main approach to detect PCa worldwide. On the other hand, TPBx was also favored by relatively fewer clinicians, owing to its potential superiority in sampling the peripheral and apical regions^7^.

In recent years, as the sextant biopsy protocol seemed to be inadequate for cancer detection, more cores have been obtained with an aim to achieve higher CDR^22^. Under this background, subsequent studies manifested that TPBx and TRBx were equivalent in CDR. Shen *et al.* performed a meta-analysis and found that there were no significant differences between the two approaches in the overall CDR and subgroup comparison^23^. Also, with saturation biopsy, evidence supported that TPBx and TRBx were both effective with a comparable CDR (31.4% vs. 25.7%)^24^. Regarding the present study, there was no significant difference between the two approaches in CDR, which was in consistent with the previous studies. Thus, it should be a general agreement that both TPBx and TRBx are effective approaches to detect PCa. On the other hand, increasing concern regarding the overdiagnosis of VLR cancer has been arisen, which is not considered as a life-threatening problem^25^. However, based on our data, the similar proportion of VLR cancer indicates that by choosing a biopsy route may not help to avoid overdiagnosis of VLR prostate cancers.

Rectum is a blood-rich organ. Previous studies suggested that the TRBx poses a higher risk of infection because the faecal carriage bacteria can easily enter the blood from sampling points^26^. In the present study, rectal bleeding and infection-related complications (including fever and sepsis) were more frequently observed in the TRBx group. Furthermore, they raised the risk of major complications in the TRBx group. Though the major complications appear to be rare, sometimes they are life-threatening. In the present study, all the patients with major complications underwent emergency therapy including endoscopic operation and blood transfusion and all received prolonged hospital treatment. According to our experience, complications of rectal bleeding or infection, no matter mild and severe, could be largely avoided by performing TPBx due to its inherent advantage in puncture route that rectum is not involved. Before deciding the biopsy procedure, it is difficult to identify the patient at high risk for rectal bleeding or infection; however, specifically, for patients with known hemorrhoids, antibiotic resistance or some other situations which may increase the risk of rectal bleeding or infection, TPBx may be a safer alternative.

On the other hand, TRBx was more timesaving. Kravchick *et al.* spent an average of only 8.45 min for each TRBx procedure^27^; however, their operating time did not include the time of DRE, disinfection, and getting up from the operating table. If adding the time for all these procedures, the total operating time was similar with the TRBx approach in the present study. The increase of operation time of TPBx is unexplained at present; however, the complex procedures of TPBx may be the key factor. In addition, we found that the relatively higher rate of sampling failure and additional anesthesia in TPBx approach brought more unexpected disruptions. Other authors also had reported this phenomenon^19^. Despite encountered at a low frequency, it could further extend the time of biopsy, and potentially influence clinicians’ choice of biopsy approach.

Based on our data, TPBx brings more pain to patients. PPNB is recommended to block the fibers located in the prostate capsule^28^ which transmits the sensation of pain during prostate biopsy, so as to provide the best pain control^29^. However, the pudendal nerve is very sensitive to the trauma from TPBx, unlike the rectal mucosa which has a lower sensitivity to the pain caused by piercing the rectal wall^30^. Therefore, the procedure of anesthesia is the primary cause of pain in patients undergoing TPBx. Moreover, the patient’s position could influence the pain level. The lithotomy position is often used in TPBx, which is more uncomfortable than the left lateral position^31^. On the other hand, most patients in the TRBx group complained about inserting the transducer; however, it was overall mild. Tüfek *et al.* evaluated the level of pain using VAS score when performing TRBx with 12-core procedure. They found that the highest VAS score was 2.95 when inserting the transducer. The other steps including anesthetic injection and sampling had a lower VAS score at about 1.56 to 2.66^32^. In another TRBx study by Kahriman *et al.*, the mean pain score was only 1.38. When asked whether the patients agreed to accept repeated biopsy, 82% of them answered “yes”^33^. On the contrary, Saredi *et al.* found the VAS score was as high as 4.09 during anesthesia when performing TPBx^34^. In addition, mild pain was seen more frequently after biopsy in the TPBx group. Evidence suggested problematic postbiopsy symptoms were linked with raised anxiety of patients^35^. Thus, the pain control during and after biopsy seem to be a critical challenge to TPBx. Therefore, the patients with low pain threshold or anxiety may be not appropriate to receive TPBx.

The study had some limitations. The first was the relatively fewer cores obtained from prostate with volume < 50 ml, which might influence the CDR and complication rate. Thus, a protocol with more cores should be conducted in further study. Secondly, the biopsy and follow-up procedures performed in a single center might differ from practices elsewhere. Therefore, a multicenter study is mandatory for evaluating the influence of different institutions. Thirdly, false negative rate could not be evaluated due to the relative short follow-up duration and low acceptance of re-biopsy among the patients. Thus, future studies in this regard are also necessary.

In summary, our prospective study supported that the CDR and overall complications rate are comparable between the TRBx group and the TPBx group, hence they are both effective approaches to detect PCa. However, it should be noted that major complications might occur more frequently in the TRBx group. On the other hand, compared with TRBx, TPBx was more complex, painful, and had a higher probability of being interrupted by the unexpected repeating biopsy or additional anesthesia. The above-mentioned findings might help the clinicians to select the appropriate biopsy approach to benefit the patients.

## Additional Information

**How to cite this article**: Guo, L.-H. *et al.* Comparison between Ultrasound Guided Transperineal and Transrectal Prostate Biopsy: A Prospective, Randomized, and Controlled Trial. *Sci. Rep.* **5**, 16089; doi: 10.1038/srep16089 (2015).

## Figures and Tables

**Table 1:** Patients’ characteristics and biopsy protocols of the TPBx and TRBx groups.

Patient characteristics and biopsy protocols	TPBx group (n = 173) No. (%)	TRBx group (n = 166) No. (%)	*P* value
Age, years	67.18 ± 6.76	67.35 ± 7.28	0.604
Body Mass Index (kg/m^2^)	25.71 ± 2.74	24.32 ± 2.62	0.398
Biospy indications			
PSA, ng/mL^a^			
median	8.81	10.48	0.858
inter-quartile range	3.6–56.0	6.2–69.0	
abnormal DRE finding	20 (11.6%)	19 (11.5%)	1.000
Ultrasound findings			
Prostate volume, ml^a^			
median	47.2	45.9	0.628
inter-quartile range	12.9–97.7	20.0–98.0	
>50 ml, 12 cores protocol	98 (54.6%)	90 (52.3%)	0.663
<50 ml, 8 cores protocol	75 (45.4%)	76 (47.7%)	0.663
With suspicious findings	40 (23.1%)	30 (20.1%)	0.284
Biopsy protocol			
Total cores	1918	1768	
Systemic biopsy cores	1776 (92.6%)	1688 (95.5%)	1.000
Additional biopsy cores	80 (4.2%)	60 (3.4%)	1.000
Failed sampling cores	62 (3.2%)	20 (1.1%)	<0.001
Fragmented	39 (2.0%)	8 (0.5%)	
<10 mm	5 (0.3%)	5 (0.3%)	
Absence	18 (1.0%)	7 (0.4%)	

Note:

^a^Data in this blank were with skewed distribution, so that presented as median and inter-quartile range and analyzed using Wilcoxon Rank-Sum test.

**Table 2:** Comparison of CDR, proportion of each pathological pattern and cancer core rate between the TPBx and TRBx groups.

Pathological diagnosis and CDR	TPBx group (n = 173) No. (%)	TRBx group (n = 166) No. (%)	*P* value
Positive	61 (35.3%)	53 (31.9%)	0.566
Adenocarcinoma	61 (35.3%)	51 (30.7%)	0.419
PSA ≤ 6	18 (10.4%)	18 (10.8%)	0.547
PSA = 7	18 (10.4%)	15 (9.0%)	1.000
PSA ≥ 8	25 (14.5%)	18 (10.8%)	0.564
Very-low-risk PCa	6 (3.5%)	5 (3.0%)	1.000
Other malignancy	0 (0%)	2 (1.2%)	0.232
Positive needles	266 (13.9%)	221 (12.5%)	0.224
Negative	112 (64.7%)	113 (68.1%)	0.566
BPH	92 (53.2%)	92 (55.4%)	0.744
PIN	13 (7.5%)	10 (6.0%)	0.668
ASAP	6 (3.5%)	8 (4.8%)	0.593
Other	1 (0.6%)	3 (1.8%)	0.363

**Note:** Abbreviation: BPH, benign prostate hyperplasia, prostatitis; PIN, prostatic intraepithelial neoplasia; ASAP, atypical small acinar proliferation.

**Table 3:** Comparison of complications rate between the TPBx and TRBx groups.

Complication rate	TPBx group (n = 167) No. (%)	TRBx group(n = 161) No. (%)	*P* value
All complications	76 (45.5%)	73 (45.3%)	0.912
Minor complications^a^	75 (44.9%)	66 (41.0%)	0.504
Mild hematuria	33 (19.8%)	37 (23.0%)	0.502
Low fever < 38.5°C	2 (1.2%)	7 (4.3%)	0.099
Mild rectal bleeding	0 (0%)	14 (8.7%)	<0.001
Mild pain	58 (35.3%)	26 (13.0%)	<0.001
Others	11 (6.6%)	12 (7.5%)	0.831
Major complications	1 (0.6%)	7 (4.3%)	0.034
Severe hematuria	0 (0%)	0 (0%)	
High fever > 38.5°C	0 (0%)	2 (1.2%)	
Sepsis	0 (0%)	1 (0.6%)	
Severe rectal bleeding	0 (0%)	2 (1.2%)	
Severe pain	0 (0%)	0 (0%)	
Vasovagal event	1 (0.6%)	2 (1.2%)	

Note:

^a^There were overlaps among different subgroups of minor complications.

**Table 4:** Comparison of operating time and VAS scores between the TPBx and TRBx groups.

Comparison	TPBx group (n = 173) No. (%)	TRBx group (n = 166) No. (%)	*P* value
Operating time, min	17.51 ± 3.33	14.73 ± 3.25	<0.001
Pain			
VAS score^a^			
median	4.0	2.0	<0.001
inter-quartile range	1.0–6.0	0.0–4.0	
Most painful procedure			
None	3 (1.7%)	37 (22.3%)	<0.001
Probe insertion	30 (14.5%)	67 (42.2%)	<0.001
Anesthesia	110 (63.6%)	29 (17.5%)	<0.001
Sampling	26 (15.0%)	25 (15.1%)	1.000
Others	9 (5.2%)	5 (3.0%)	0.415
Additional anesthesia, number of times	26 (15.0%)	2 (1.2%)	<0.001

Note:

^a^Data in this blank were with skewed distribution, so that presented as median and inter-quartile range and analyzed using Wilcoxon Rank-Sum test.
